# Evaluating a German learning disorders platform using the RE-AIM framework

**DOI:** 10.1016/j.heliyon.2024.e39968

**Published:** 2024-10-30

**Authors:** Lior Weinreich, Gido Metz, Björn Witzel, Olga Hermansson, Paula Dümig, Gerd Schulte-Körne, Kristina Moll

**Affiliations:** a Department of Child and Adolescent Psychiatry, Psychosomatics and Psychotherapy, University Hospital, LMU Munich, Nussbaumstrasse 5, 80336, Munich, Germany; b Care and Public Health Research Institute, Faculty of Health, Medicine and Life Sciences, Maastricht University, Universiteitssingel 40, 6229 ER, Maastricht, the Netherlands

**Keywords:** Online platform evaluation, Learning disorders, Matomo analytics, User experience

## Abstract

In recent years, online platforms have made educational, medical, and other professional content easily accessible, but research evaluating such platforms is still scarce. The purpose of the current study is to evaluate LONDI, a German learning disorders platform. The platform offers scientifically based information for different user groups, and an algorithm-based help system that professionals can use to facilitate diagnosing and planning interventions. The evaluation is focused on the user group of learning therapists using the platform and its help system. It is theoretically grounded on the RE-AIM framework and assesses four of its dimensions: Reach, Adoption, Implementation and Maintenance. Results from an online questionnaire (*N* = 496) showed that the platform reaches a large proportion of learning therapists. Another online questionnaire (*N* = 150) revealed that most users say they would adopt the help system, and this is predicted by its pragmatic qualities. Data from the Matomo web analytics software (*N* = 8,459 online visits) displayed diverse patterns in the platform’s implementation. Future research is needed to further examine their meaning in the context of health-related education. Web analytics also revealed that usage patterns are not maintained. Rather, there is an increase in the number of users and in smartphone usage over time, coinciding with a decrease in the average time spent on the platform. Consequently, future efforts will be dedicated to optimizing smartphone compatibility. This study is the first to utilize the RE-AIM framework with web analytics, paving the way for further theory-grounded platform evaluations.

## Highlights

• The rise in digitalization merits more theory-based online platform evaluations.
• This evaluation uses RE-AIM and several methods including Matomo web analytics.
• User experience measurements reveal that usage is predicted by pragmatic qualities.
• Path conversion and abandonment rates vary and need to be interpreted contextually.
• Increases in users and smartphone usage coincide with a decrease in usage time.

## Graphical abstract

**Figure:**
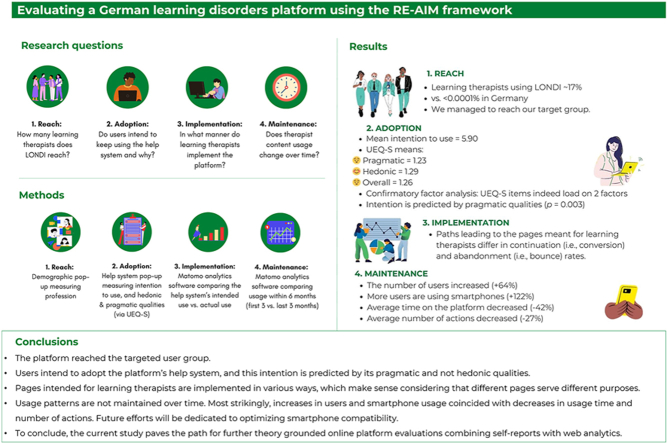

## 1 Introduction

In recent years, the rise in digitalization has made it both possible and advisable for printed content to become easily accessible online This possibility is also being leveraged for health-related educational content, made widely available through online platforms [1]. In turn, online platforms necessitate research evaluating if they are indeed effective (e.g., [[2], [3]]).

### 1.1 LONDI: a German learning disorders platform

The present study aims to evaluate LONDI, a German learning disorders platform (www.londi.de). Learning disorders are generally defined as persistently lagging academic skills and performance [4]. Worldwide, 5–15 % of all children fulfill the diagnostic criteria for a learning disorder (American Psychiatric Association, 2013; Horowitz et al., 2017). Specifically, affected children suffer from deficits in reading, spelling, and or arithmetic. Adding to this difficulty, they are also likely to suffer from other comorbid disorders (e.g., ADHD; [5]). This, in turn, contributes to their susceptibility to social difficulties (e.g., bullying; [6]). Importantly, learning disorders are not explained by intellectual disabilities, poor auditory or visual abilities, limited motivation, or limited possibilities to acquire education. Moreover, they are accounted by both genetic and environmental factors [7]. When children with learning disorders try and fail to master basic academic skills and do not receive appropriate help, they are likely to get frustrated and either act out or give up [8]. Therefore, it is crucial to provide them with appropriate support.

To support children with learning disorders, the LONDI platform was tailored to the needs of five different user groups interacting with affected children: parents, learning therapists, teachers, school psychologists and social workers. LONDI provides both user specific information and support. In terms of information, LONDI provides scientifically based content about specific learning difficulties. The information is different for each user group to best accommodate for their specific needs (e.g., only the section for social workers includes a breakdown of the law on assistance integration). In terms of support, LONDI includes parental coaching and an algorithm-based help system for professionals (e.g., learning therapists).

The help system serves as a tool to identify the appropriate diagnostic or intervention recommendations for each child, according to their individual learning profile. To this aim, prior to the current study, possible diagnostic and intervention tools were assessed based on scientific quality criteria. The quality criteria included: theoretical basis, objectivity, reliability, validity and unambiguity. Accordingly, recommendations were classified using a star system (e.g., one star means criteria were not met, and three means all criteria were met). Moreover, prior to the current study, each recommendation was classified according to four competence levels. The competence levels were founded on widely used developmental models for literacy [9,10] and arithmetic skills [11,12]. These developmental models encompass age-appropriate skill acquisition and competencies. The four competence levels used in the help system indicate what each recommendation covers for every learning skill. The first competence level refers to basic skills, the second to initial skills, the third to standard skills, and the fourth to applied and flexible skills. The priorly assessed and classified diagnostic and intervention tools serve as a database for the help system. When a professional uses the help system, an algorithm uses this database to match the data they insert with the most suitable recommendations. Along with the name and information for each recommendation, the help system also displays which competence levels the recommendation covers, the sample size and year in which its norms were calculated and its scientific quality rating. For experienced learning therapists, this process takes approximately 5 min per recommendation. A detailed breakdown of the help system steps is appended to this manuscript.

### 1.2 Online evaluation research

To date, online evaluation research varies considerably. Whereas some research focused on data from various user analytics matrices to evaluate online behavior (e.g., [[13], [14], [15], [16], [17]]), other research combined user analytics data with various self-report measures (e.g., [[18], [19], [20], [21], [22], [23], [24], [25]]). Notably, a recent evaluation by Merkt et al. [26] combined user log files, behavioral data from a laboratory study, and objective content characteristics. Likewise, we sought to combine different methods for an encompassing evaluation, which we grounded on a theoretical framework. Namely, the RE-AIM framework.

### 1.3 RE-AIM framework

RE-AIM is a theoretical framework often used for evaluating public health interventions [27,28]. As suggested by Glasgow et al. [28], it is optimal to use mixed-methods while using the RE-AIM framework. RE-AIM assesses five dimensions: Reach, Effectiveness, Adoption, Implementation and Maintenance. *Reach* addresses the proportion of the target population an intervention reaches (e.g., [[29], [30]]); *Effectiveness* addresses an interventions’ success rate in terms of its impact on individual outcomes (e.g., [31]); *Adoption* addresses the proportion of users that adopt an intervention (e.g., [32]); *Implementation* addresses the manner in which an intervention is applied in real-life settings (e.g., [33]); and *Maintenance* addresses the manner in which an intervention is used over time (e.g., [34]).

### 1.4 Web analytics

A suitable method for evaluating an online platform is using web analytics (e.g., [35]). While the most prominent web analytics software is Google Analytics, Matomo is a software that provides an optimal alternative, compatible with European data ownership and privacy law [36,37]. Matomo’s features provide insight on the behavior of online users via anonymous visitor profiles and various matrices including users' location and software. Moreover, Matomo includes features to evaluate usage via pre-defined objectives. The pre-defining of objectives can include specified goals (e.g., pages you want users to visit), and funnels (i.e., the steps users go through on their way to the goals).

Although Matomo and other web analytics software are still predominantly used for commercial and marketing purposes [14], in recent years, there has been a shift towards utilizing web analytics for scientific research. Several studies have used Matomo to evaluate online health related educational content (e.g., [[17], [19], [20], [22], [23], [35]]). At present, however, it seems that none have used the RE-AIM framework [27] as a theoretical backbone. Arguably, given the plethora of evaluation studies grounded on RE-AIM in various settings for over two decades [28], and its recent usage in several notable digital platform evaluations (e.g., [[38], [39], [40], [41]]) it can be deemed a suitable framework for the current study. Thus, generating more research utilizing web analytics for theory grounded scientific evaluation research.

### 1.5 The current study: hypotheses

The current study’s goal is to evaluate LONDI using the RE-AIM framework. It was pre-registered as part of a larger evaluation project. This project contains two more pre-registered studies that differ from the current study in their focus, sample, theoretical background and methods [42,43]. Specifically, the other studies use a sample of recruited participants, as well as a mixed-method approach containing both quantitative and qualitative measures. In contrast, the current study was framed within a real-world setting of anonymous online users, using LONDI of their own volition (i.e., not as part of an experiment). Due to this real-world setting, the current study uses a solely quantitative approach. The current study’s hypotheses were grounded on four of the RE-AIM dimensions: Reach, Adoption, Implementation and Maintenance. The study does not include the Effectiveness dimension since there was no feasible measurement for intervention success based on Matomo data (e.g., improvement in therapeutic outcomes). This is in line with past research that only focused on the RE-AIM dimensions within their scope of feasibility (e.g., [[29], [33]]). Moreover, Glasgow et al. [28] recommended to only focus on the RE-AIM dimensions compatible with a study’s hypotheses and within its scope. Comparable with other web evaluations, we focused on a homogeneous user group experienced with developmental learning disorders, and on specific pages or sections (e.g., [17]). Namely, we focused on learning therapists, their designated pages, and the evaluation of the help system.

In line with the official RE-AIM website [44], which defines Reach as the absolute number, proportion, or representativeness of users, we formulated the first hypothesis. Hypothesis 1: The relative percentage of learning therapists using the platform will be higher than their percentage in the general German population. Moreover, in line with the RE-AIM scoring instrument [45] and the official RE-AIM website [44], which define Adoption as the number of users willing to use a program, and encourage an evaluation of the qualities of a program that influence Adoption, we formulated the second hypotheses. Hypothesis 2a: Users will state that they intend to keep using the help system. Hypothesis 2b: Users will rate the system as above average in terms of pragmatic and hedonic qualities. Hypothesis 2c: The pragmatic and hedonic ratings will predict the intention to use the help system. Additionally, considering the official RE-AIM website [44], which defined Implementation as the percentage of achieved process objectives, we formulated the third hypothesis. Hypothesis 3: The platform will be fully implemented by learning therapists reaching it via all possible online paths (i.e., links). Lastly, in line with the official RE-AIM website [44], which defined Maintenance as the assessment of how and why a program did or did not prosper over long-term usage, we formulated the fourth hypothesis. Hypothesis 4: Therapist content usage will change over time, as indicated by comparing platform usage in different time-segments (i.e., 01.01.23–31.03.23 vs. 01.04.23–30.06.23).

## 2 Methodology

### 2.1 Sample

The sample consisted of *N* = 8,459 LONDI visits that took place in the six months between 01.01.23 and 30.06.23. Beforehand, approval was obtained from the ethics committees in all collaborating institutions. Moreover, before answering any of the questionnaires, users were asked to give informed consent, in accordance with the Declaration of Helsinki. Most visits originated from search engines (*n* = 5,707) and direct entries (*n* = 2,296). Others originated from links in other websites (*n* = 393) and social media posts (*n* = 63). Moreover, most visits were from Germany (*n* = 6,684) and among these mostly from the city Frankfurt am Main (*n* = 3,912). Notably, the relatively large sample from Frankfurt am Main can be explained by the fact that one of the partner universities working on LONDI and advertising the platform is located there. Among others, visits were also from Offenburg (*n* = 355), Munich (*n* = 257) and Düsseldorf (*n* = 132). Furthermore, most visits were done via smartphones (*n* = 5,129), followed by desktops (*n* = 2,991). The average visit duration was 2 min and 15 s.

### 2.2 Measures

To test the hypotheses for each of the RE-AIM dimensions, the following operationalizations and assessments were planned.

#### 2.2.1 Reach

**Fig. 1:**
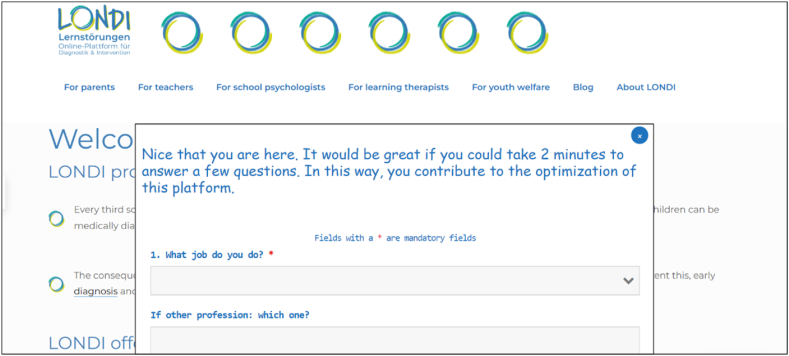
Screenshot of the demographic pop-up questionnaire appearing in the front page. *Note.* The demographic pop-up questionnaire and the front page that appear in this screenshot were translated from German to English for the benefit of non-German speaking readers.

Operationalization for hypothesis 1 (Reach: percentage of learning therapists using the platform): Data on users' profession was gained via an optional demographic pop-up questionnaire, filled out voluntarily by a sub-sample of the platform users. Completing the questionnaire takes 2 min at most. Moreover, it appears to all first-time users on the front page (see Fig. 1). Possible answers are the German equivalents of: a. teacher, b. school psychologist, c. learning therapist, d. no profession/unemployed, e. other. This was used to determine what percentage of the users visiting the platform are learning therapists. Thus, the percentage of learning therapists among all other professions indicated in the pop-up was calculated. Then, it was compared to the percentage of learning therapists in the entire German population.

#### 2.2.2 Adoption

Operationalization for hypothesis 2 (Adoption): Data was collected via the German equivalent of the item: “I plan to continue using LONDI” that appears in an optional pop-up questionnaire in the help system, and was taken from a validated questionnaire [46]. Possible answers are the German equivalents of: a. Do not agree at all, b. Do not agree, c. Strongly disagree, d. Neutral, e. Tend to agree, f. Agree, g. Fully agree. At present, there are no benchmarks for the used item in the context of health interventions and online platforms. We therefore used the mean user rating of the answers on a 7-point scale (1 = Do not agree at all to 7 = Fully agree) as cutoff to define adoption. Namely, if participants scored above the mean of 4, we consider it as an indication for intention to adopt the help system. In previous research, this item was often used in combination with other items to gain deeper understanding of underlying usage mechanisms (e.g., [[47], [48]]). This was also done in the current study as described in the following passage.

To gain a deeper understanding of why users intend to keep using the help system or not, the same pop-up questionnaire contained the short version of the User Experience Questionnaire, assessing the pragmatic and hedonic qualities of the help system (UEQ-S; [49]). Completing the UEQ-S takes 2 min at most. The UEQ-S is widely used to measure how users subjectively experience a product (e.g., [50]), and includes benchmarks for interpretation (see Table 1). The UEQ-S includes eight items assessing the help system’s hedonic and pragmatic qualities (i.e., four items for each), on a 7-point scale. Each item is represented by two opposite terms. Moreover, they are scaled from −3 (i.e., the most negative answer) to +3 (i.e., the most positive answer), with 0 representing a neutral answer. All German UEQ-S items used for the study, as well as the English version are appended to this manuscript. Below are examples of one pragmatic and one hedonic item, respectively:

**Table 1:** Mean User Experience Questionnaire (UEQ-S) ratings of the current study compared to Benchmarks retrieved from Hinderks et al. [51].

		Benchmarks				
Qualities	Mean rating	Excellent	Good	Above average	Below average	Bad
Pragmatic	1.23	>1.73	1.55–1.73	1.15–1.54	0.73–1.14	<0.73
Hedonic	1.29	>1.55	1.25–1.55	0.88–1.24	0.57–0.87	<0.57
Overall	1.26	>1.58	1.4–1.58	1.02–1.39	0.68–1.01	<0.68

I find the LONDI help system:

complicated o o o o o o o easy

boring o o o o o o o exciting.

For both examples, the utmost left circle is scaled as −3, and the utmost right circle as +3.

#### 2.2.3 Implementation

Operationalization for hypothesis 3 (Implementation): Data on how the learning therapist user group implements the platform was collected using the Matomo analytics software. For this, we used the goals and funnels features, which must be pre-defined. The goals are defined as target pages one wants users to visit. The funnels are defined as different paths users can take to reach these goals. Once these are defined, data is collected revealing users' entry points and drop-offs during their journey across the platform toward the pre-defined goals. By defining goals and funnels, it is possible to determine where one loses visitors in converting one’s goals. In londi.de, the three pages intended for learning therapists are.

1 For learning therapist page
2 Information for learning therapist page
3 Help system

For the sake of brevity, from this point forward, the front page is referred to as “Front,” the for learning therapists page as “Index,” the help system page as “Help System” and the information for learning therapists page as “Information.” The aim of the Front page is to serve as an index page used to direct learning therapists to other pages. Thus, it does not contain a lot of content, with its primary goal to help users find what they are looking for (e.g., a link to detailed information on learning therapy). In contrary, the other two pages are goal pages, and contain detailed information (e.g., information on who finances learning therapy). For the present study, we have defined the goals as reaching the above-mentioned pages meant for learning therapists, and the funnels as the different paths learning therapists can take to reach these pages. To reach any of the above-mentioned pages, there are six possible sequences.

Subsequently, the six paths were defined as follows.

1. Front - > Index
2. Front - > Help System
3. Front - > Index - > Help System
4. Front - > Information
5. Front - > Index - > Information
6. Front - > Information - > Help System

During the data collection period, the number of users completing or not completing these paths was analyzed, also known as conversion and abandonment rates, respectively. Path abandonment in this context is comparable with bounce rates, a term often used in online marketing to refer to the percentage of online visits that end after just one pageview [14]. Acceptable bounce rates are highly dependent on the specific web page, its goals, and its contents. At present, there are no benchmarks for bounce rates in the context of online platforms related to learning disorders, but rather from the realm of online marketing. Such benchmarks for marketing campaigns [52] are as follows.

• 25 % or lower: Bad – there might be a technical issue underlying such low rates since most websites' bounce rate range is between 26 and 70 %.
• 26–40 %: Excellent.
• 41–55 %: Average.
• 56–70 %: Higher than normal but could make sense if the content fulfills the users' needs. and they do not feel the need to look at other pages afterwards.
• 70 % or higher: Bad – there might be a technical issue underlying such high rates such as slow loading time, a misleading title, or content that is not smartphone friendly.

#### 2.2.4 Maintenance

Operationalization for hypothesis 4 (Maintenance): Data on platform usage in different time-segments (i.e., 01.01.23–31.03.23 vs. 01.04.23–30.06.23) was compared using the Matomo analytics software. Specifically, the number of users using LONDI in the first three months was compared with the last three to see whether the number has increased, remained as it was, or decreased. Furthermore, we compared: users’ locations, time spent on the platform in general and on specific pages, number of actions, times of the day in which the platform was being used, and the devices and software used.

### 2.3 Analyses

Matomo indices were used to analyze online user behavior, within a six-months period (i.e., 01.01.23–30.06.23). Furthermore, a confirmatory factor analysis was run to assess whether the UEQ-S items indeed load on two factors (i.e., hedonic and pragmatic). Lastly, multiple linear regression (MLR) was used to find out whether the help system’s hedonic and pragmatic qualities predict intention to use (i.e., the answer to the statement “I plan to continue using LONDI”).

## 3 Results

### 3.1 Reach

To test hypothesis 1 (Reach): Data on users' profession was collected via an optional demographic pop-up questionnaire that appears to all first-time users on the front page. Notably, we only included learning therapists that used LONDI of their own volition. A total of *N* = 496 visitors answered the questionnaire. Among these, 17.53 % (*n* = 87) reported that they are learning therapists (*n* = 85 females). In terms of experience: 10 % (*n* = 9) were self-proclaimed novices; 23 % (*n* = 20) had 1–5 years of professional experience; 24 % (*n* = 21) had 6–10 years of professional experience; 29 % (*n* = 25) had 10–20 years of professional experience; and 14 % (*n* = 12) had more than 20 years of professional experience. The 18+ population in Germany in 2022, and the above 19+ population in Austria in 2023 were approximately 70 and 7 million, respectively [53,54]. Within the German population, the number of learning therapists is not publicly regulated. However, based on correspondence with the German Association for Dyslexia & Dyscalculia (BVL), it is estimated that the combined number in Germany and Austria (there are no separate estimates for the countries) is 2,500 [55]. Given this estimate, their proportion in the adult German population is smaller than 0.0001 %. This supports hypothesis 1, that the relative percentage of learning therapists using the platform is higher than their percentage in the general German population.

### 3.2 Adoption

To test hypothesis 2 (Adoption): Data was collected via the item: “I plan to continue using LONDI” that appears together with the UEQ-S in an optional pop-up questionnaire in the help system. A total of *N* = 150 visitors answered the questionnaire. The item was rated on a 7-point scale (1 = Do not agree at all to 7 = Fully agree). The mean rating was 5.90. As there are no benchmarks for the single item evaluation, and since this mean is above 4, we consider it as an indication for intention to adopt the help system. This supports hypothesis 2a, stating that users intend to keep using the help system. To compare the results with the UEQ-S scores detailed below, we re-coded the answers from −3 (i.e., for the most negative answer) to +3 (i.e., for the most positive answer), with 0 representing a neutral answer, resulting in a mean rating of 1.90.

We then calculated the mean answers to the UEQ-S items assessing the pragmatic and hedonic qualities of the help system. Due to a programming error, the UEQ-S was rated on an 8-point scale instead of 7. To correct this, the scale was transformed. Namely, the two middle points were merged (as they represent the neutral options), and we calculated the other answers as intended from −3 (i.e., the most negative answer) to +3 (i.e., the most positive answer), with 0 representing the neutral answer. The mean scores and their interpretation according to the benchmarks are: 1.23 for the pragmatic qualities, which is above average, and 1.29 for the hedonic qualities, which is good. The overall score was 1.26, which is above average (see Table 1). This supports hypothesis 2b, stating that users will rate the system as above average in terms of pragmatic and hedonic qualities.

Moreover, a confirmatory factor analysis (CFA) was performed to assess whether the UEQ-S items indeed load on two factors (i.e., hedonic and pragmatic). Each factor was indicated by the hedonic and pragmatic variables based on Schrepp et al. [49]. The CFA was performed using the lavaan package in R (version 4.3.0). The CFA model fit was evaluated using the Comparative Fit Index (CFI), Tucker-Lewis Index (TLI), Root Mean Square Error of Approximation (RMSEA), and Standardized Root Mean Square Residual (SRMR). The fit indices for the model were as follows: CFI = 0.91; TLI = 0.85; RMSEA = 0.14; SRMR = 0.06. Overall, the model shows a moderate to good fit [56].

Additionally, a multiple linear regression (MLR) was used to find out whether the help system’s hedonic and pragmatic qualities predict intention to use via the answers to the statement “I plan to continue using LONDI.” The model was statistically significant, *F*(2,156) = 24.94, *p* < .001, and explained 23 % of the variance. While the pragmatic qualities had a significant positive effect on the intention to use, *t*(156) = 3.00, *p* = .003, it was not statistically significant for the hedonic qualities *t*(156) = 1.97, *p* = .051. This only partially supports hypothesis 2c, stating that pragmatic but not hedonic ratings will predict the intention to use the help system.

### 3.3 Implementation

To test hypothesis 3 (Implementation): Data using the Matomo goals and funnels features was collected. We defined the goals as reaching the pages meant for learning therapists, and the funnels as the different paths learning therapists can take to reach them. Thereafter, path continuation (i.e., conversion) and abandonment rates were calculated and evaluated according to bounce rate marketing benchmarks ([52]; see Table 2). Notably, within the sample of *N* = 8,459 LONDI visits, *n* = 3,108 and *n* = 1,326 were visits to the help system and the learning therapists pages, respectively.

**Table 2:** *Data on path conversion and* abandonment *rates*

Path	Conversion Rate	Abandonment Rate	Interpretation according to bounce rate benchmarks
1. Front - > Index	68.6 %	31.4 %	Excellent (26–40 %)
2. Front - > Help System	55.9 %	44.1 %	Average (41–55 %)
3. Front - > Index - > Help system	34.7 %	65.3 %	Higher than normal (56–70 %)
4. Front - > Information	91.6 %	8.4 %	Bad (25 % or lower)
5. Front - > Index - > Information	25.5 %	74.5 %	Bad (70 % or higher)
6. Front - > Information - > Help System	48.5 %	51.5 %	Average (41–55 %)

*Note*. Bounce rates benchmarks retrieved from Willson ([52]). Benchmark range is indicated within parentheses. Front = front page; Index = for learning therapists page; Help System = help system page; Information = information for learning therapist page.

As seen in Table 2, three of the six paths had good abandonment rates, ranging between average and excellent according to marketing benchmarks. Specifically, abandonment rates for path 1 (i.e., Front - > Index); path 2 (i.e., Front - > Help System); and path 6 (i.e., Front - > Information - > Help System) were excellent, average, and average, respectively.

Notably, abandonment rates for path 4 (i.e., Front - > Information) were relatively low (i.e., 8.4 %) and considered bad. In the context of marketing, this might be interpreted as a technical problem. However, in the context of LONDI, low abandonment rates might indicate that the page served as the visitors' goal. This is in line with this page’s purpose, which is to give users detailed information rather than refer them to other pages (i.e., the opposite of an index page). Thus, this might indicate that the purpose of many LONDI learning therapist users is to get detailed information via this page.

In contrast, abandonment rates for path 5 (i.e., Front - > Index - > Information) were relatively high (i.e., 74.5 %) and are also considered bad. Since each additional step adds an additional possibility to abandon the path, and this path includes three steps, by default abandonment rates would be higher than the path with only the first two steps (i.e., path 1). Moreover, it could be that experienced learning therapists did not enter the information page because they are already familiar with the relevant information, so instead they turned immediately to other pages or left the platform.

Similarly, for path 3 (i.e., Front - > Index - > Help System) the abandonment rates were higher than normal (i.e., 65.3 %). In this case, since the help system requires time and input from the users, it could be that learning therapists did not enter the help system unless they had enough time to insert the details of a child in mind.

All in all, these results only partially support hypothesis 3, stating that the platform will be fully implemented by learning therapists reaching it via all possible online paths (i.e., links).

### 3.4 Maintenance

To test hypothesis 4 (Maintenance): Data on platform usage in different time-segments (i.e., 01.01.23–31.03.23 vs. 01.04.23–30.06.23) was compared using the Matomo analytics software. The results are shown below in Table 3. These results support hypothesis 4, stating that therapist content usage will change over time, as indicated by comparing platform usage in different time-segments (i.e., 01.01.23–31.03.23 vs. 01.04.23–30.06.23). Within the sample of *N* = 8,459 LONDI visits, *n* = 3,202 and *n* = 5,257 were in the first and the second time segment, respectively. Notably, we only included Germany and Austria in this table as most users were situated in these countries. Likewise, we only included the most used devices (i.e., desktops and smartphones), and software (i.e., Windows, Mac, iOS, Android). We found that in the first time-segment (i.e., 01.01.23–31.03.23) most visits per local time were between 09:00–19:00 (i.e., 76 %); and that in the second time-segment (i.e., 01.04.23–30.06.23) most visits per local time were between 09:00–21:00 (i.e., 79.6 %).

**Table 3:** Platform usage in different time-segments

Matrix	T1	T2	Difference (%)
Number of users	3.202	5,257	64 %
Users located in Germany	2,483 (77.5 %)	4,201 (79.9 %)	69 %
Users located in Austria	83 (2.6 %)	101 (1.9 %)	22 %
Avg. time spent on platform	3 min 2s	1 min 46s	−42 %
Avg. time spent on help system	1 min 10s	30s	−57 %
Avg. time spent on therapists info	52s	1 min 28s	69 %
Avg. number of actions	4.4	3.2	−27 %
Time of the day with most visits	14:00 (8.6 %)	14:00 (7.6 %)	0 %
Visits via desktops	1,455 (45.4 %)	1,536 (29.2 %)	6 %
Visits via smartphones	1,592 (49.7 %)	3,537 (67.3 %)	122 %
Visits via Windows	737 (23 %)	814 (15.5 %)	10 %
Visits via Mac	398 (12.4 %)	375 (7.1 %)	−6%
Visits via iOS	298 (9.3 %)	811 (15.4 %)	172 %
Visits via Android	276 (8.6 %)	748 (14.2 %)	171 %

*Note.* T1 and T2 stand for the first and second time-segments: 01.01.23–31.03.23 and 01.04.23–30.06.23, respectively.

## 4 Discussion

The present study aimed to evaluate a German learning disorders platform, by testing hypotheses based on four of the RE-AIM framework dimensions (i.e., Reach, Adoption, Implementation and Maintenance). Specifically, the evaluation focused on the pages for learning therapists and on the help system section of the LONDI platform. Notably, the LONDI platform was developed to combat the educational difficulties and psychological stress affecting children with persistent learning disorders. To this aim, the platform supports relevant user groups by providing information and resources to recognize learning difficulties as early as possible, and for professionals to find effective supporting measures. The platforms' user specific content and algorithm-based help system set LONDI apart from other sources. However, in the context of the German speaking population interested in learning disorders, valuable online resources could be found elsewhere. For example, the websites of the Federal Association for Dyslexia and Dyscalculia [57], and the professional association for integrative learning therapy [58]. Nevertheless, the current study’s findings support the notion that LONDI adds a valuable contribution in this context. This is supported, for instance, by the relatively large number of learning therapists using LONDI of their own volition, hence, potentially, leading learning therapists to integrate the platform in daily practice (e.g., routinely opening the information for learning therapists page to retrieve the LONDI protocol sheet for meetings with parents). Moreover, the study has implications for future evaluations in other contexts, as it paves the way for more studies to utilize web analytics for theory-grounded evaluation research. Insights concerning each RE-AIM dimension are detailed below.

### 4.1 Reach

With regards to hypothesis 1 (Reach), we found that LONDI managed to reach a large proportion of learning therapists (17.53 %), that is much greater than the proportion in the general German population (<0.0001 %). A similar Reach evaluation was utilized by Fuller et al. [41], measuring the number of users using a digital health tool. Nevertheless, as outlined by Lee et al. [29], retaining reach to specific user groups may be challenging, and requires continuous resources and effort. Future efforts will be devoted to continuously attract learning therapists to use LONDI (e.g., via online marketing). Consequently, future evaluations should examine whether LONDI’s learning therapists' reach is retained over longer periods of time.

### 4.2 Adoption

With regards to hypothesis 2 (Adoption), we found that most users intend to continue using the help system. However, we acknowledge that this finding should be interpreted cautiously. Firstly, intention to use was measured by a single self-reported item to make the questionnaire as brief as possible, and it is based on a relatively small sample (*N* = 150). Consequently, it is possible that there was a bias. Namely, that different aspects of the intention to use were lost due to brevity, or that only those interested to continue using the help system answered the item. Secondly, users answered the questionnaire after using the help system but before receiving its recommendations. The placement before the recommendations was deliberate, chosen to maximize the number of users choosing to fill out the questionnaire before leaving the help system. However, it is possible that this placement influenced ratings. Nevertheless, valuable insights regarding Adoption could be deduced. Specifically, we found that users rate the help system as above average in terms of its pragmatic qualities (e.g., how efficient it is), and as good in terms of its hedonic qualities (e.g., how interesting it is). Future efforts will be devoted to further improve the pragmatic qualities (e.g., make the help system less complicated). Interestingly, when we checked if the pragmatic or hedonic qualities predict intention to use, we found that only pragmatic qualities predict it. Similarly, Merkt et al. [26] showed that when it comes to online educational videos, adoption is related to their content and not their features (i.e., film cuts). Thus, the results suggest that pragmaticism is a key motivator for online educational and professional usage. This further supports our decision to try to improve the help system’s pragmatic qualities.

### 4.3 Implementation

With regards to hypothesis 3 (Implementation), we found that paths leading to the pages meant for learning therapists differ in continuation (i.e., conversion) and abandonment rates. Among the six examined paths, two of the paths had bad abandonment rates (i.e., too low or high) according to marketing benchmarks [52]. Notably, path 5 had the highest abandonment rates (74.5 %). It could be that the high abandonment rates were related to the fact that the path has more than two steps. However, they could also be related to potential technical barriers. Future efforts will be devoted to further investigate this path and improve possible technical issues impacting the abandonment rates (e.g., increasing smartphone friendliness). Nevertheless, since the benchmarks were taken from the marketing realm, these abandonment rates might not be bad when examined in the context of the platform. A learning disorders platform such as LONDI is likely to have different standards than a commercial website. Adequate implementation should be assessed in light of an intervention’s or in this case a platform’s purpose, as advocated by Yardley et al. [59] and Metz et al. [17]. Moreover, high abandonment rates could indicate that a page is not relevant, or rather that users have sufficient knowledge and the content is no longer necessary [59]. Additionally, abandonment rates should be interpreted differently for different pages within the same website. For example, a page solely containing links to other pages with the goal of directing people (e.g., ‘are you looking for information on Dyscalculia’) might have higher abandonment rates than pages with detailed information. That might have been the case in path 3, which had an abandonment rate of 65.3 % (see Table 2). This path contained the index page, a page meant to direct learning therapists to detailed information or to the help system. Its abandonment rate is the highest compared to paths 2 and 6 that also lead to the help system either from the front page or the information page, with rates of 44.1 % and 51.5 %, respectively.

Interestingly, the maintenance measurements comparing the first three with the last three months revealed a decrease in the average time users spent on the help system (i.e., 1 min 10s vs. 30s). Conversely, there was an increase in the time spent on the information for learning therapists page (i.e., 52s vs. 1 min 28s). The time needed to use the information page is hard to approximate, as it includes various sub-sections that can be read separately. However, for the help system, it can be approximated that for experienced learning therapists, the process of filling out the required fields takes about 5 min per recommendation. As the average time spent on the help system is much lower, especially in the last three months, it could be that learning therapists left the help system without using it. It is also possible that visits to the help system were made for the purpose of finding out what information is needed to get a recommendation for a specific child in mind (e.g., test results).

Furthermore, to better understand if users actually read the content or use the help system, future efforts will also be devoted to setting time goals. Specifically, a goal will be timed depending on how long it should take users to use a feature or read content, based on average word per minute reading speed [60]. If this time elapsed, not counting the time a page is minimized, a goal counts as complete (i.e., a conversion). For example, a time goal for using the help system will count as a conversion if users have stayed in the help system for more than 3 min. Conversely, a time goal for using the information pages will already count as a conversion if users have stayed on the page for more than 1 min. All in all, further research examining abandonment rates for theory-grounded scientific evaluations is needed, and should be interpreted contextually.

### 4.4 Maintenance

With regards to hypothesis 4 (Maintenance), several interesting findings emerged. On the one hand, when we compared the number of users using LONDI in the first three vs. the last three months, we saw an increase of 64 % in the number of users (i.e., 3.202 vs. 5,257). This is somewhat encouraging, and stands apart from other evaluation studies that found an inverse maintenance pattern (e.g., [34]). On the other hand, when we compared the average time spent on the platform and number of actions, we saw a decrease of 42 % and 27 %, respectively. A possible explanation could be that many of the visits in the last three months were by returning users. Since Matomo keeps users’ IP addresses anonymous, we could not test this assumption. Nevertheless, it is likely that returning users spend less time on the platform as they are familiar with its content and structure. A future evaluation could add an item in the demographic questionnaire, asking users if they have used the platform before. An alternative or additional explanation could be that many of the visits in the last three months were done by smartphones (i.e., 49.7 vs. 67.3 % in the first and last three months, respectively). According to Kamerer [14], smartphone visits are typically shorter than desktop visits. These visits usually have a specific purpose, and users are less inclined to do more content exploration. Moreover, when pages are not well suited for smartphone usage, users are more likely to get frustrated and leave a page. Therefore, future efforts will be devoted to further optimize LONDI for smartphone usage.

### 4.5 Limitations

The current study is not without limitations. Firstly, as underlined by Howe et al. [20], it is impossible to know from web analytics if users actually read content. Thus, it is impossible to know from our Matomo data if learning therapists read the content in their designated information page. However, considering the relatively low abandonment rates from this page, it is likely that its content was regularly read. Moreover, future endeavors will be devoted to setting time goals based on average word per minute reading speed [60], to examine if users surpass the minimum required time to read a page. Secondly, due to Matomo’s compliance with strict data protection laws, all users remain anonymous. This means that it is not possible for us to deduce which of the users are first time users, and which are returning users. Moreover, the current study’s users did not participate in an experiment nor shared their contact information. Thus, it was not possible to incorporate qualitative complementing methods such as the mixed-method approach by Metz et al. ([61]; e.g., incorporating a thinking aloud method). Nevertheless, the other parts of the larger LONDI evaluation project provide complementing data (e.g., via interviews; [[42], [43]]). Moreover, the various matrices collected by Matomo (e.g., average time spent on the platform in different time periods) and our additional questionnaires (e.g., asking users if they intend to use the help system again) provide valuable insights and a foundation for further improvements. Thirdly, due to limited resources, platform users could not interact with a live person (e.g., via a live chat). Future efforts will be devoted to creating a conversational agent (Chatbot; [62]) as a means of providing user support and measuring user engagement.

## 5 Conclusion

The current study evaluated the German learning disorders platform LONDI. The platform evaluation was grounded on the RE-AIM framework, and utilized questionnaires and web analytics. Overall, the study’s results revealed that the LONDI platform adds a valuable contribution to German speaking learning therapists. Specifically, the platform reached a large proportion of the targeted user group of learning therapists. Future efforts will be dedicated to examine whether this reach is retained over time. Moreover, users reported that they intend to adopt the platform’s help system, and this intention was predicted by its pragmatic but not by its hedonic qualities. Future efforts will be dedicated to further improve the help system’s pragmatic qualities. Additionally, we saw that the pages intended for learning therapists were implemented in various ways, as indicated by their conversion and abandonment rates. Notably, this interpretation was based on marketing research, which has a different context than that of the current evaluation, and future efforts are needed to further investigate usage in a health-related educational context, and to examine whether users spend enough time on specific pages to count them as read. Finally, we saw that usage patterns are not maintained over time. Most strikingly, increases in users and smartphone usage coincided with decreases in usage time and number of actions. This could be attributed to the fact that smartphone visits are typically shorter than desktop visits. Alternatively, it could be that over time users return to the platform for shorter visits. Future efforts will be dedicated to optimizing smartphone compatibility, and investigating the proportion of returning users. Overall, while it was evident that the platform reached the learning therapists user group, and that they intend to use it, further investigations and improvements are needed. The current study provides a basis for informed policy and funding decisions regarding a health-related educational platform. Furthermore, it paves the path for further theory-grounded online platform evaluations, combining self-reports with web analytics.

## CRediT authorship contribution statement

**Lior Weinreich:** Writing – review & editing, Writing – original draft, Visualization, Software, Methodology, Investigation, Formal analysis, Data curation, Conceptualization. **Gido Metz:** Writing – original draft, Methodology, Conceptualization. **Björn Witzel:** Conceptualization. **Olga Hermansson:** Conceptualization. **Paula Dümig:** Conceptualization. **Gerd Schulte-Körne:** Supervision, Resources. **Kristina Moll:** Writing – review & editing, Supervision, Methodology, Conceptualization.

## Data and code availability

Data will be made available on request.

## Ethics statement

This work was approved by the ethics committee at the university hospital LMU Munich [approval number 22–0300 1 V].

## Funding

This work was supported by the 10.13039/501100002347Federal Ministry of Education and Research [grant numbers 01GJ2101A, 01GJ2101B].

## Declaration of competing interest

The authors declare the following financial interests/personal relationships which may be considered as potential competing interests:

Gerd Schulte-Koerne reports financial support was provided by 10.13039/501100002347Federal Ministry of Education and Research Bonn Office. If there are other authors, they declare that they have no known competing financial interests or personal relationships that could have appeared to influence the work reported in this paper.
