# Supplementary material for: Evaluating a German learning disorders platform using the RE-AIM framework
Source: Heliyon. 2024 Oct 30;10(21):e39968. doi: 10.1016/j.heliyon.2024.e39968 (PMC11566692; doi:10.1016/j.heliyon.2024.e39968)
Supplement: Multimedia component 1 [file mmc1.docx]

# Appendix

**Table A1**

*Items of the German short version of the User Experience Questionnaire (UEQ-S)*

| Negative item | Rating | Positive item |
| --- | --- | --- |
| behindernd | o o o o o o o | unterstützend |
| kompliziert | o o o o o o o | einfach |
| ineffizient | o o o o o o o | effizient |
| verwirrend | o o o o o o o | übersichtlich |
| langweilig | o o o o o o o | spannend |
| uninteressant | o o o o o o o | interessant |
| konventionell | o o o o o o o | originell |
| herkömmlich | o o o o o o o | neuartig |

**Table A2**

*Items of the English short version of the User Experience Questionnaire (UEQ-S)*

| Negative item | Rating | Positive item |
| --- | --- | --- |
| obstructive | o o o o o o o | supportive |
| complicated | o o o o o o o | easy |
| inefficient | o o o o o o o | efficient |
| confusing | o o o o o o o | clear |
| boring | o o o o o o o | exciting |
| not interesting | o o o o o o o | interesting |
| conventional | o o o o o o o | inventive |
| usual | o o o o o o o | leading edge |

**Figure A1**

*Screenshot of the first help system page*

*
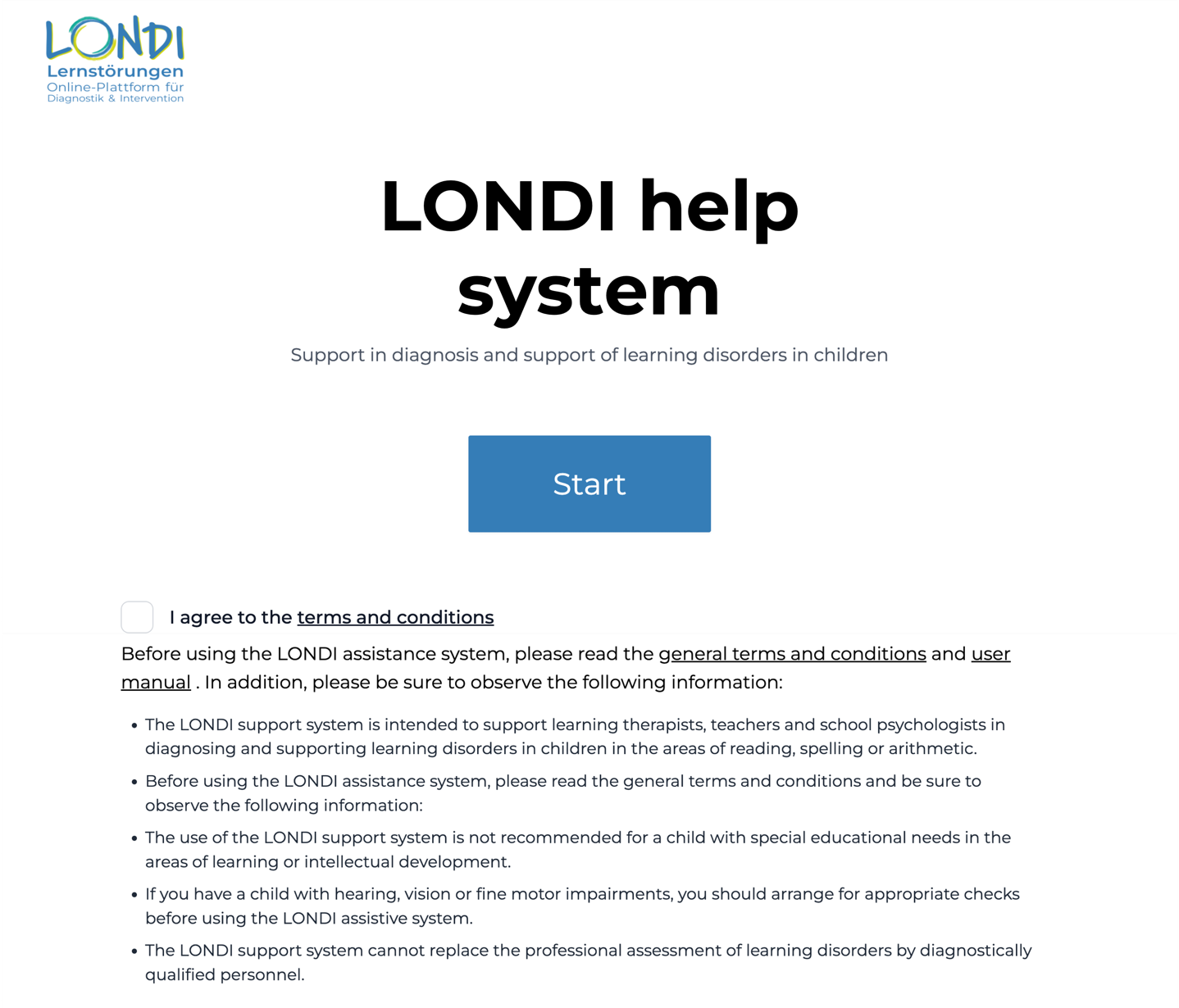
*

Help with diagnosing and supporting learning disorders in children

*Note.* This page and those following were translated from German to English for the benefit of non-German speaking readers. Since the translation was done using the Google Chrome auto-translation function, some terms referring to the help system appear interchangeably. This page details the terms and conditions for the use of the help system.

**Figure A2**

*Screenshot of the second help system page*

*
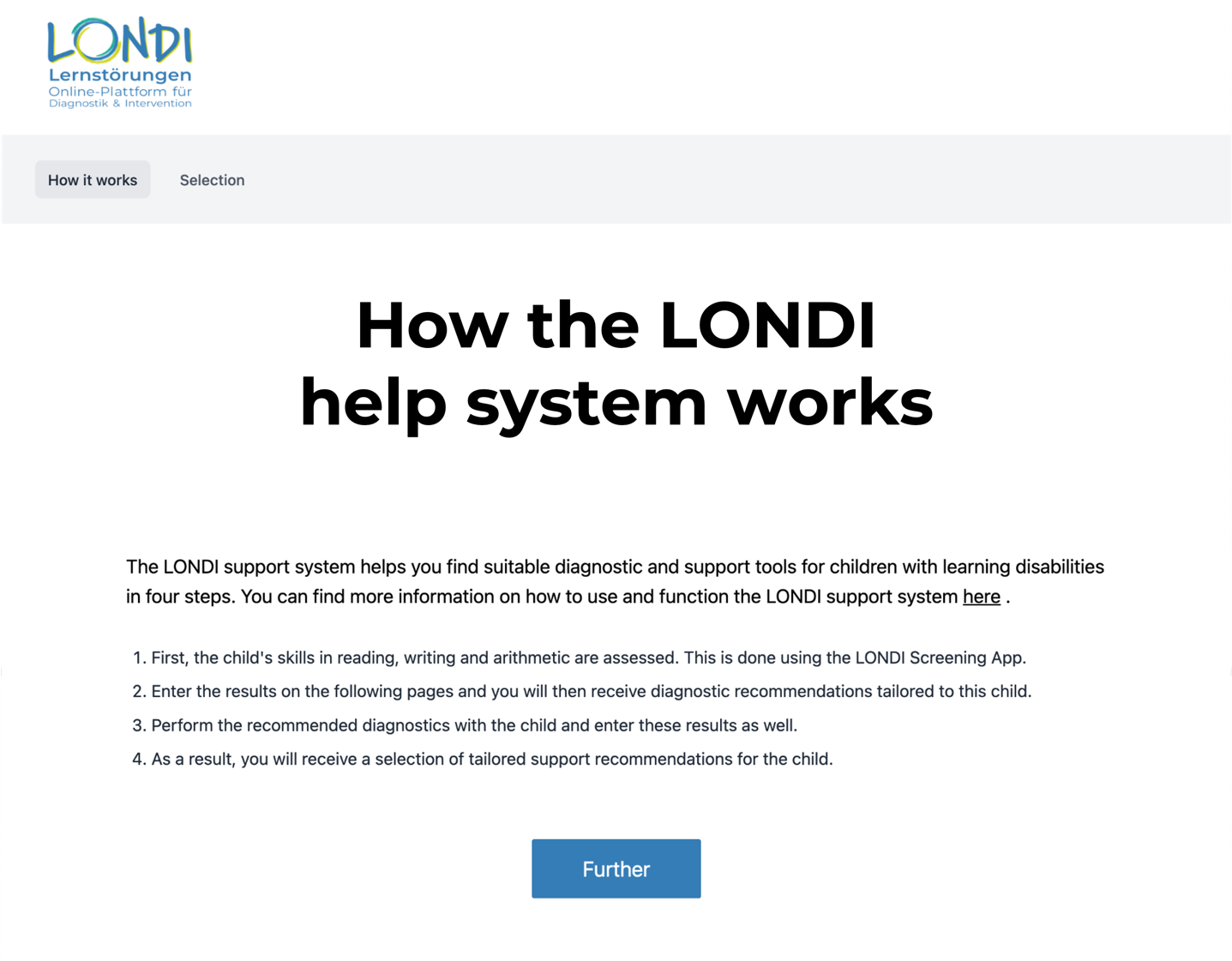
*

*Note.* This page explains how the help system works. As detailed in the first step, professionals are instructed to use an additional screening app to assess a child’s learning skills. However, it is also possible for them to skip this step and instead use their professional judgment.

**Figure A3**

*Screenshot of the third help system page*

*
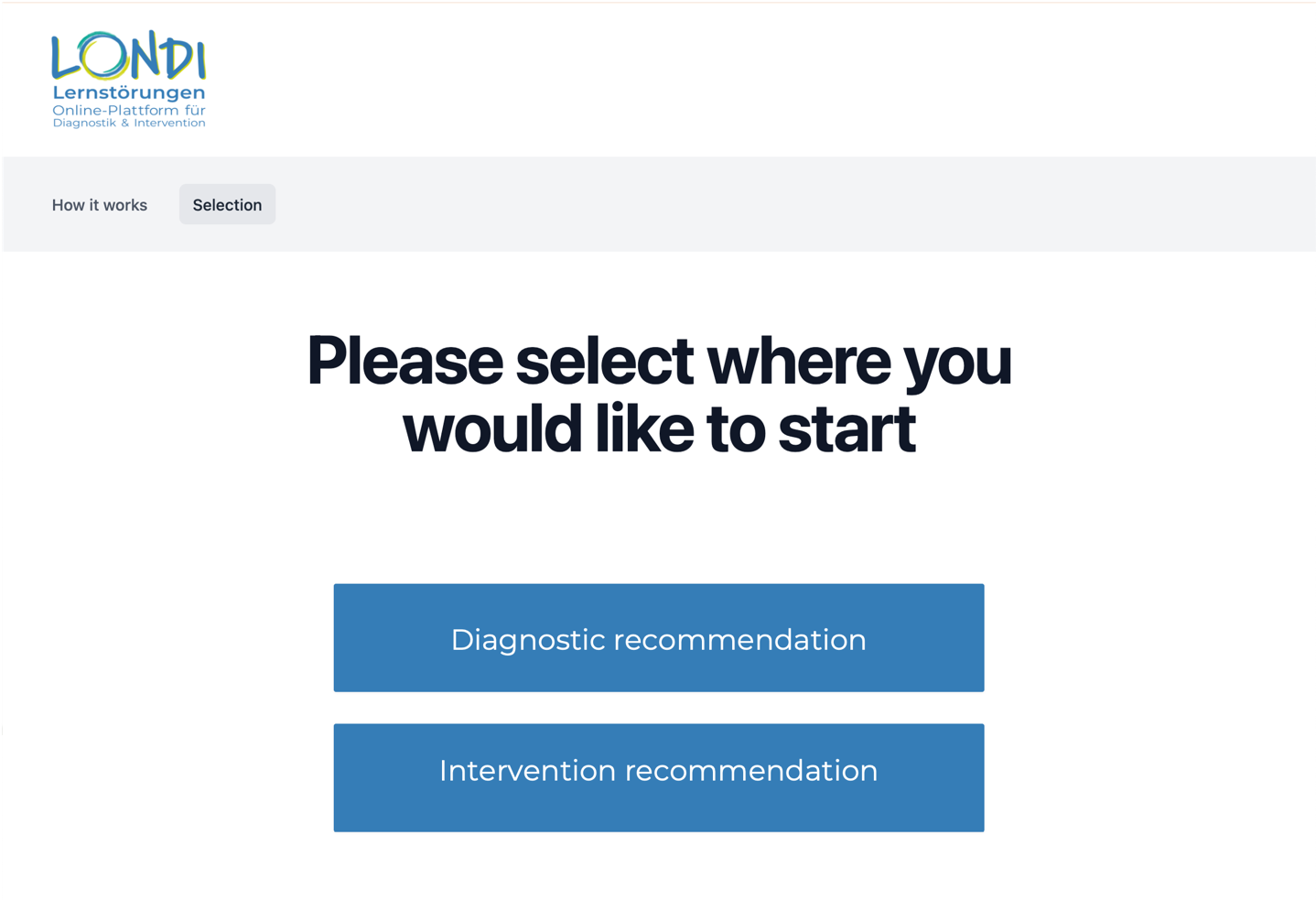
*

*Note.* On this page, professionals choose between diagnostic and intervention recommendations.

**Figure A4**

*Screenshot of the fourth help system page after choosing “Diagnostic Recommendation”*


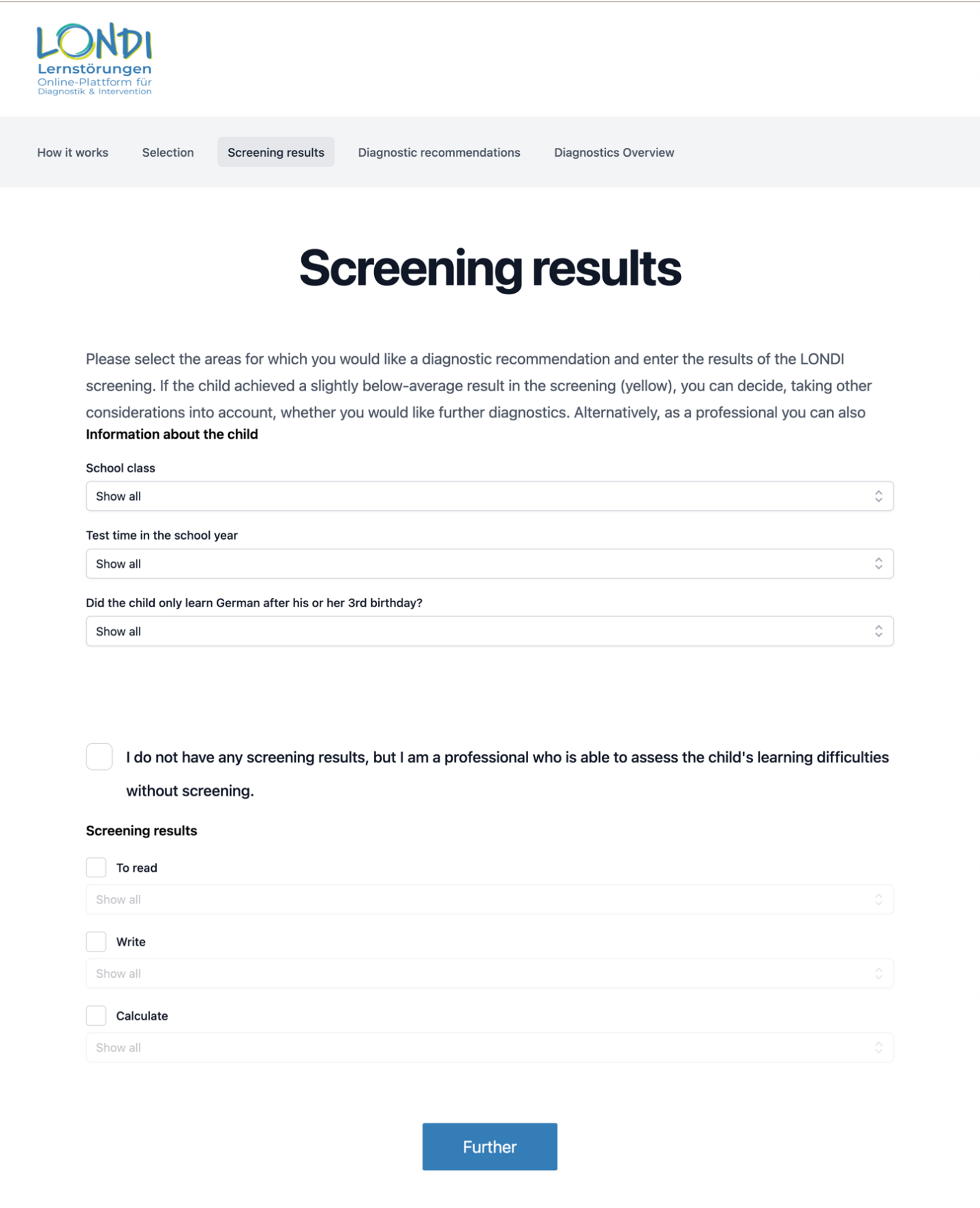


Please select the areas for which you would like a diagnostic recommendation and enter the results of the LONDI screening. If the child has achieved a slightly below-average result in the screening (yellow), you can decide whether you would like further diagnostics, taking other considerations into account. Alternatively, as a professional, you can also assess the areas in which you suspect learning difficulties.

*Note*. On this page professionals fill-out the child’s data. It includes a language acquisition question to determine whether German was learned as a secondary language.

**Figure A5**

*Screenshot of an example of the fourth help system page with filled-out data*


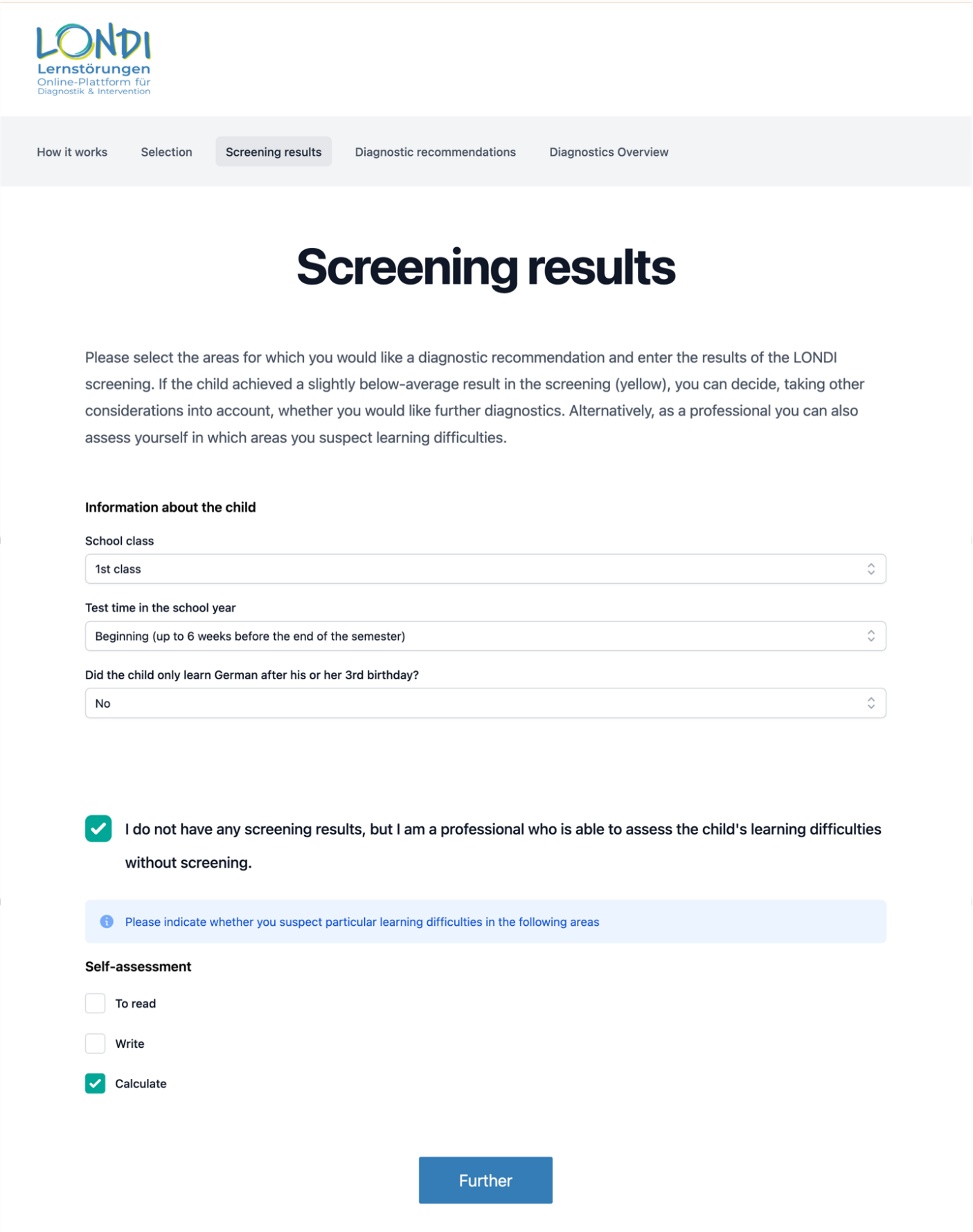


Please select the areas for which you would like a diagnostic recommendation and enter the results of the LONDI screening. If the child has achieved a slightly below-average result in the screening (yellow), you can decide whether you would like further diagnostics, taking other considerations into account. Alternatively, as a professional, you can also assess the areas in which you suspect learning difficulties.

*Note*. This example indicates that the child is in first grade, in the beginning of the school year, learned German before their third birthday, and that they have difficulties in arithmetic.

**Figure A6**

*Screenshot of the fifth help system page*


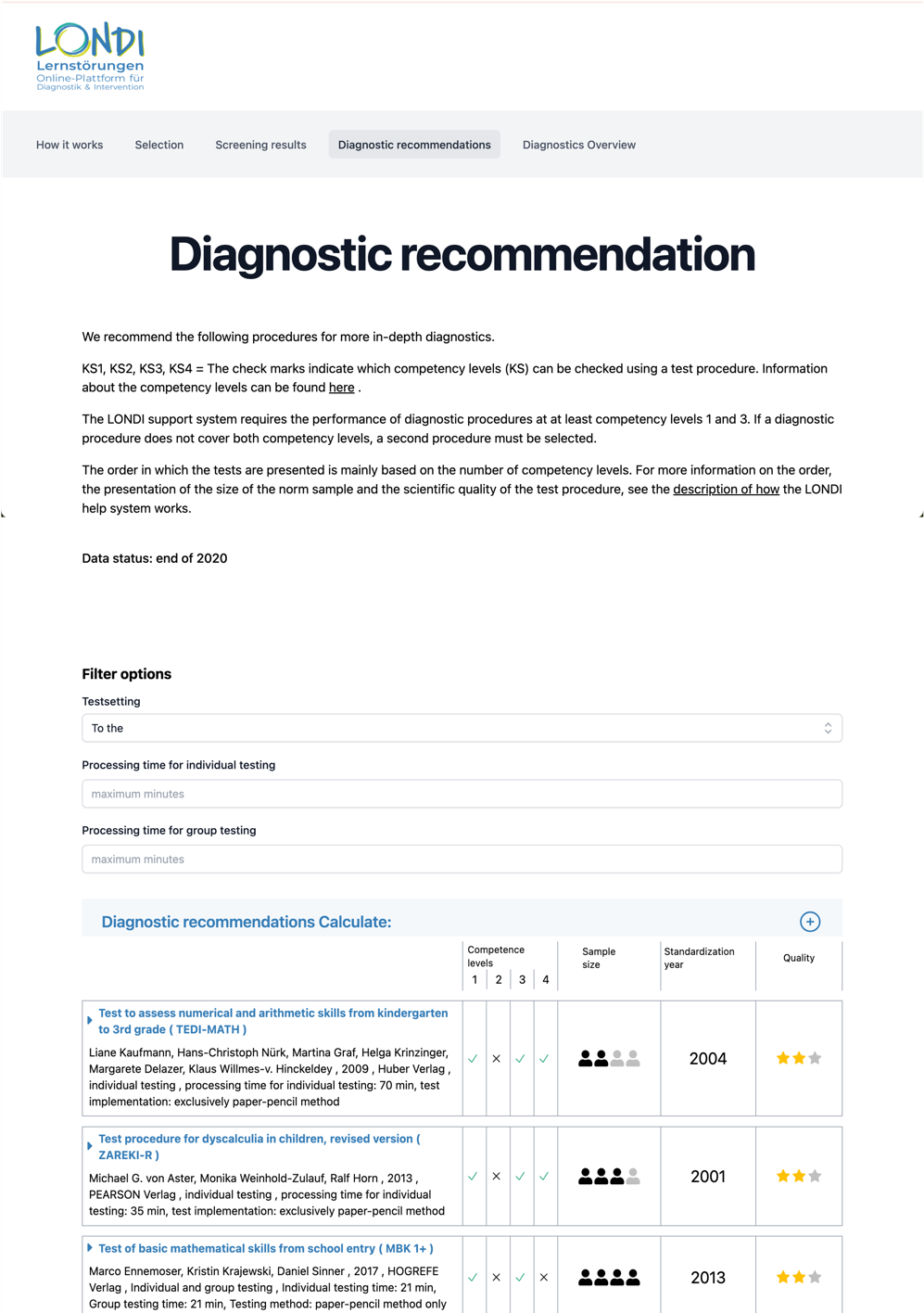


*Note.* This page suggests individualised diagnostic recommendations.

**Figure A7**


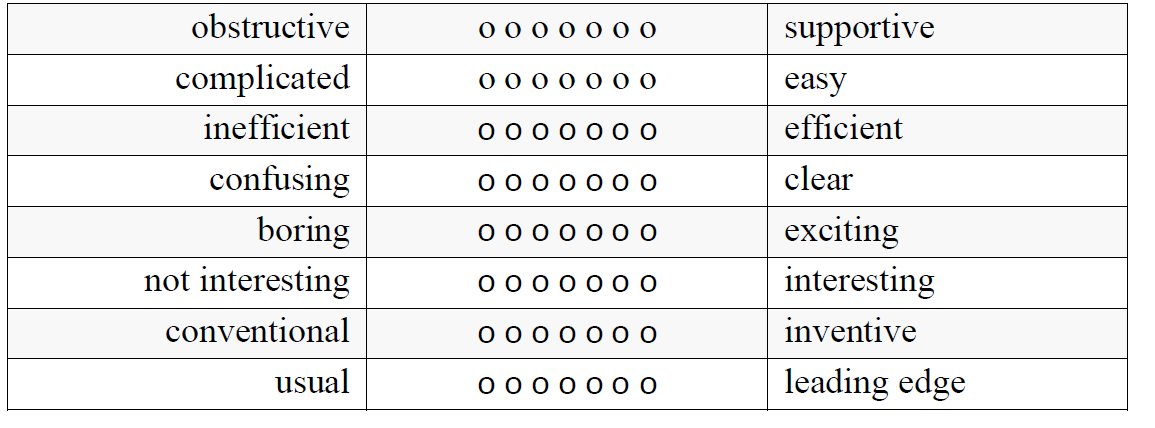
*Screenshot of the pop-up questionnaire following the fifth help system page*


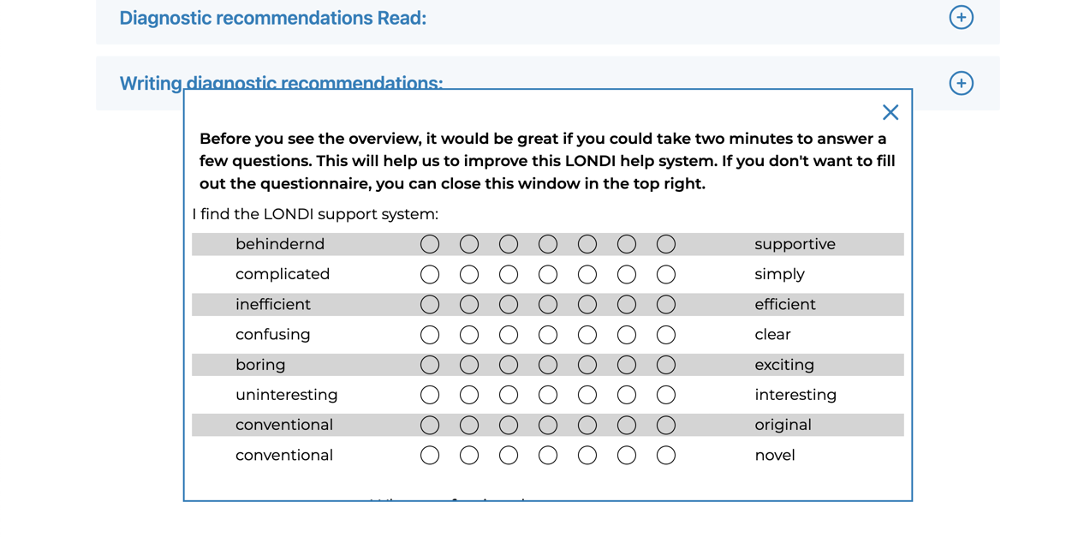


**Figure A8**

*Screenshot of the sixth and final help system page*

**
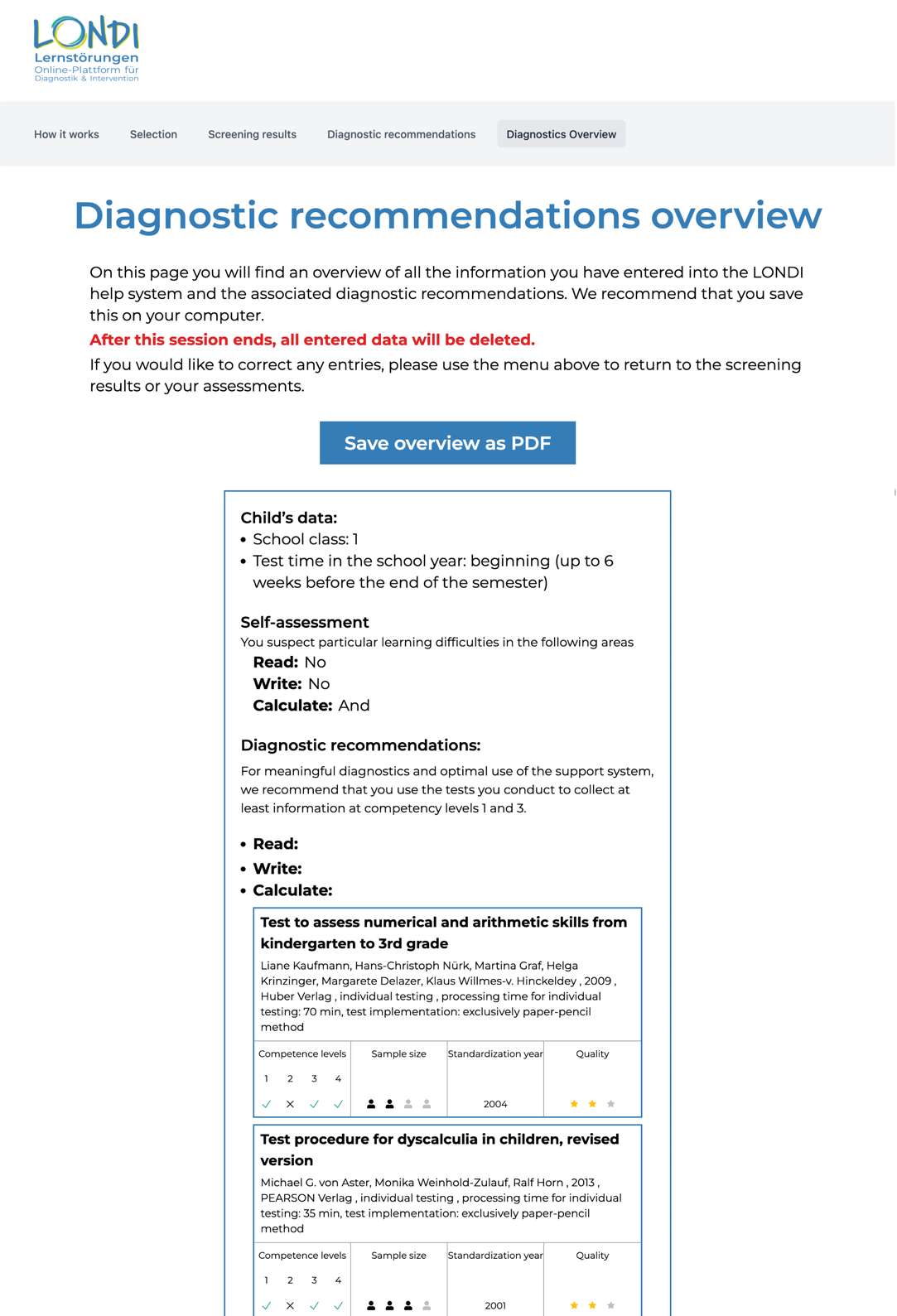
**

Yes

*Note.* This page summarizes the diagnostic recommendations. As data cannot be saved in the platform due to data protection regulations, professionals have the option to download the page as a PDF file.
